# Mathematical model of broadly reactive plasma cell production

**DOI:** 10.1038/s41598-020-60316-8

**Published:** 2020-03-03

**Authors:** Samantha Erwin, Lauren M. Childs, Stanca M. Ciupe

**Affiliations:** 1 Oak Ridge National Laboratory, Biomedical Sciences, Engineering, and Computing Group, Oak Ridge, TN 37830 USA; 2 Virginia Tech, Mathematics, Blacksburg, Virginia 24061 USA

**Keywords:** Immunology, Mathematics and computing

## Abstract

Strain-specific plasma cells are capable of producing neutralizing antibodies that are essential for clearance of challenging pathogens. These neutralizing antibodies also function as a main defense against disease establishment in a host. However, when a rapidly mutating pathogen infects a host, successful control of the invasion requires shifting the production of plasma cells from strain-specific to broadly reactive. In this study, we develop a mathematical model of germinal center dynamics and use it to predict the events that lead to improved breadth of the plasma cell response. We examine scenarios that lead to germinal centers that are composed of B-cells that come from a single strain-specific clone, a single broadly reactive clone or both clones. We find that the initial B-cell clonal composition, T-follicular helper cell signaling, increased rounds of productive somatic hypermutation, and B-cell selection strength are among the mechanisms differentiating between strain-specific and broadly reactive plasma cell production during infections. Understanding the contribution of these factors to emergence of breadth may assist in boosting broadly reactive plasma cells production.

## Introduction

Upon infection or vaccination, somatically hypermutated high-affinity antibodies specific to antigenic challenge are produced by long lived plasma cells inside secondary lymphoid organs, called germinal centers^1,2^. The somatic hypermutation process is initiated by antigenic activation of B-cell clones that undergo several rounds of affinity-driven selection, proliferation, and somatic hypermutation^3–5^. The number of B-cells seeding the germinal center was believed to be as low as three "founder” B-cells, whose variability was lost in the process of selection, which favored the offsprings with the highest affinity for the antigen^6–8^. New experimental results, using multiphoton microscopy, have determined that germinal centers are seeded by hundreds of "founder” B-cells from different B-cell clones. Over time, the clonal diversity is maintained or lost at different rates in different germinal centers^9^.

Understanding the mechanistic interactions that favor the evolution of predominantly homogeneous or heterogeneous germinal centers is the main purpose of our study. It is known that the development, selection, and diversification of B-cell clones inside germinal centers is done under strict competition for survival signals provided by T-follicular helper cells (Tfh) and antigen-presenting follicular dendritic cells^10,11^. B-cells circulate between the light and dark zones of the germinal centers. In the light zone they bind to and present antigen peptide-MHC complexes to Tfh-cells, which in turn provide help and survival signals to B-cells. Selected B-cells migrate into the dark zone where they undergo proliferation and somatic hypermutation^4,12^. This process, called homogenizing selection, occurs in an iterative manner with Tfh-cells signaling believed to be the regulating factor in both the size and the composition of germinal centers. Homogenizing selection promotes the survival of B-cell clones with the highest affinity for antigen^4,12–14^. Recent studies showed that selection does not always favor a single B-cell clone, and instead heterogeneous germinal centers composed of multiple B-cell clones with equal or different affinities to antigen may be the outcome^9,15^. It was hypothesized that the homogeneity of the germinal centers requires rapid selection and expansion early on of B-cells from the same clone^9^. By contrast, heterogeneity and polyclonality of germinal centers occurs stochastically and may be induced by the type of antigenic challenge, the persistence of an infection, and/or the limitation in Tfh-cell numbers needed for selection.

The selective role of Tfh-cells in the evolution of germinal centers is particularly visible during chronic virus infections, where as many as 70–90 rounds of B-cell somatic hypermutations may occur^16^. In HIV infections, for example, Tfh-cell frequencies are higher in patients that develop broadly neutralizing antibodies^17–19^. The affinity of B-cells for conserved HIV peptides is low, therefore a broadly neutralizing antibody producing germinal center needs to select for lower affinity B-cell clones. It is known that the germinal centers are populated with Tfh-cells with different antigen specificities, and that Tfh-cells migrate between different germinal centers, potentially providing survival signals to B-cells of different affinities^11^, and therefore generating polyclonality. Tfh-cells are themselves under regulation from T follicular regulatory cells, which limit their number and prevent the expansion of autoreactive cells^20^. Additionally, upregulation of programmed cell death proteins (e.g. PD-1) on Tfh-cells and their corresponding ligands on the surface of B-cells results in decreased Tfh-cell expansion, especially in the case of chronic virus infections^21^. Finally, under chronic viral challenge, Tfh-cells may be abnormal and dysfunctional and/or prolonged exposure to antigen may lead to their exhaustion^22^. Understanding Tfh-cells’ contribution to the selection of B-cell clones under stress is still under investigation and will be a driving force for our study.

Mathematical modeling has long been used to study immune responses during infection with several investigations specifically focusing on germinal center evolution. Early efforts to model germinal centers utilized agent-based strategies and found that B-cell clonal competition in germinal centers for T-cell help is a driving factor in B- cell clone selection^23^. Other studies characterized the selection of B clones by Tfh-cells, as well as subsequent B-cell division, based on successful antigen availability^24^. More recently, a stochastic model was created to provide insight into the evolution of germinal centers using birth-death-mutation processes^1^. In the context of HIV, it has been investigated how broadly-reactive B-cell clones are out-competed by lineage-specific B-cell clones^25–28^. All of these modeling efforts have provided insight into germinal center selection and maturation, yet the role of Tfh-cells in the dynamics of germinal centers seeded by more than one type of B-cell clone has yet to be characterized mathematically.

Accordingly, we propose a mathematical model for the interaction of B-cells and Tfh-cells. Our previous work, which considered the development of a germinal center seeded by cells of one B-cell clone, showed that Tfh-cells are a limiting resource in driving large numbers of somatic hypermutations^29^. We presented possible mechanisms, such as the presence of unlimited Tfh-cell help, that can revert this limitation in the presence of non-mutating and mutating antigen^29^. In this study, we investigate how the dynamics of a germinal center change when seeded by cells from two different B-cell clones. We are interested in determining under what conditions germinal centers favor dominance of plasma cells produced by a single B-cell clone versus those produced by two B-cell clones, and how this is related to the breadth of the immune response.

We consider two B-cell clones and their non-antigenic stimuli, in the form of Tfh-cell populations. One B-cell clone has weak specificity for the challenging antigen, as it responds to a rapidly mutating antigen with a lower probably of selection. However, it receives broad survival signals from more than one Tfh-cell family (for simplicity we only consider two such Tfh-cell families). To be clear, B-cells processing the same antigen may present more than one epitope to Tfh-cells, eliciting support from more than one Tfh-cell family. We refer to this type of B-cell clone as broadly reactive. The other B-cell clone uses just its clone-specific Tfh-cell population. For example, this clone may respond to a conserved epitope and thus be selected more rapidly. We refer to this clone as strain-specific. Such a scenario describes a chronic infection with a mutating virus, where plasma cells produced by the strain-specific B-cell clone will have a strong initial effect that the virus will mutate away from, while the plasma cells produced by the broadly reactive B-cell clone would have a continuous long-term, yet weak, effect. We aim to determine whether the specificity or the breadth of the B-cell clones for Tfh-cell signals is favored for survival, strength and/or breadth of the plasma cell responses following the germinal center reaction.

### Model Formulation

We consider B-cells, *B*_*j*,*i*_, with *j* ∈ {1, 2} representing the clone and *i* ∈ {0, 1, . . , *n*} representing the stage of selection (see Box 1). Cells of each B-cell clone undergo rounds of selection when presented with antigenic stimulation and Tfh-cell help. We consider that each Tfh-cell family consists of two subpopulations: *G*_*j*_ and *H*_*j*_ for *j* ∈ {1, 2}, corresponding to the availability, or lack thereof, of Tfh-cell help for B cells selection. We assume B-cells of the first clone, *B*_1,*i*_, receive broad, but weak, survival signals from both available Tfh-cell subpopulations, *G*_1_ and *G*_2_, respectively. We refer to ${\{{B}_{1,i}\}}_{i=0}^{n}$ as the broadly reactive B-cell clone. By contrast, B-cells of the second clone, *B*_2,*i*_, receive strong survival signals from a single available Tfh-cell subpopulation, *G*_2_, for which they have high specificity. We refer to ${\{{B}_{2,i}\}}_{i=0}^{n}$ as the strain-specific B-cell clone. Since the exact timing of plasma cells output by a B-cell clone is incompletely understood, we assume it occurs following *n*_*c*_ = 2∕3 ⋅ *n* stages of somatic hypermutation regardless of the type of B-cell clone producing it.

We do not model recruitment of Tfh-cells, whose initial number is given by fixed initial conditions, *G*_1_(0) = *G*_2_(0), *H*_1_(0) = *H*_2_(0) = 0. We assume that the available Tfh-cell level is at its maximum capacity at the start of the simulations and transition between available and unavailable populations. The initial population is strictly composed of available Tfh cells for B-cell selection, but later transition to the unavailable class. Cells from both Tfh-cell subpopulations *G*_*j*_ and *H*_*j*_ are lost through natural death at per capita rate *d*_*G*_. It is known that the overall Tfh-cell number is regulated by immunological processes. In particular, T follicular regulatory cells which co-localize with Tfh-cells, control their expansion and modulate the Tfh-cell role in somatic-hypermutation^20^. Additionally, upregulation of PD-1 on Tfh-cells and of its corresponding ligand PD-L1 on B-cells results in decreased Tfh-cell expansion, especially in the case of chronic virus infections^21^. We neglect the details of Tfh-cell control, and instead assume that the overall Tfh-cell magnitude is limited by B-cell populations, which compete for Tfh-cell stimulation. We model this as the transition of available Tfh-cells into the unavailable class at rates $\eta {\sum }_{i=0}^{n}{B}_{1,i}$ for the *G*_1_ population and $\eta {\sum }_{i=0}^{n}{B}_{1,i}+\eta {\sum }_{i=0}^{n}{B}_{2,i}$ for the *G*_2_ population, where *η* is the loss of availability rate of the Tfh-cells for B-cells selection. This is a reversible process, with unavailable Tfh-cells becoming available at rates $f{\sum }_{i=0}^{n}{B}_{1,i}$ for the *H*_1_ population and $f{\sum }_{i=0}^{n}{B}_{1,i}+f{\sum }_{i=0}^{n}{B}_{2,i}$ for the *H*_2_ population, where *f* is the regain of availability rate of the Tfh-cells for B-cells selection. If we assume that *η* ≤ *f*, we will observe a reduction over time in the Tfh-cell population’s availability for B-cell clone selection.

We model germinal center dynamics as follows. An initial number of B-cells of each clone, *B*_*j*,0_(0) (for *j* ∈ {1, 2}), seed the germinal center. Under antigenic stimuli and Tfh-cell help B-cells undergo up to *n* stages of somatic hypermutation. We assume four different events may happen during each stage *i* of somatic hypermutation: a forward mutation with probability *p*, a deleterious mutation with probability *d*, a neutral mutation with probability *q*, and a backwards mutation with probability 1 − *p* − *d* − *q*. The transitions from stage *i* to stages *i* + 1, *i* − 1 and *i* occur at selection rate *σ* or *σ*_*c*_ as follows. The total selection rates for cells in the strain-specific and broadly reactive B-cell clones are *σG*_2_ and *σ*_*c*_(*G*_1_ + *G*_2_), respectively. During each stage of selection, class *B*_*j*,*i*_ (for *j* ∈ {1, 2}) increases by *pα*_*i*_*B*_*j*,*i*−1_ offspring obtained through forward somatic-hypermutations for *i* ∈ {0, …, *n*}; and by (1 − *p* − *q* − *d*)*α*_*i*_*B*_*j*,*i*+1_ offspring obtained through backwards somatic-hypermutations for *i* ∈ {0, …, *n* − 1} (((1 − *p* − *q* − *d*)*α*_*n*_*B*_*j*,*n*_, for *i* = *n*, respectively); or it either remain in the same class at rate *qB*_*j*,*i*_ or die at rate *dB*_*j*,*i*_, for *i* ∈ {0, …, *n*}. Here, the expansion rate *α*_*i*_ increases by an equal percent during each forward selection stage *i*. All B-cells, *B*_*j*,*i*_, regardless of stage, die at the same per capita rate *d*_*B*_. Note that we do not explicitly incorporate antigen, whose presence is assumed in the chosen rates *σ*. Lastly, we assume that plasma cells *P*_*j*_ (*j* ∈ {1, 2}) are produced at fixed rate *κα*_*k*_ by all B-cell clones *B*_*k*_ that have reached *k*-rounds of somatic hypermutations, *n*_*c*_ ≤ *k* ≤ *n*.

The system describing these interactions is given by:

1 $$\begin{array}{ccc}\frac{d{G}_{1}}{dt} & = & -{d}_{G}{G}_{1}-\eta {G}_{1}{\sum }_{i=0}^{n}{B}_{1,i}+f{H}_{1}{\sum }_{i=0}^{n}{B}_{1,i},\\ \frac{d{H}_{1}}{dt} & = & -{d}_{G}{H}_{1}+\eta {G}_{1}{\sum }_{i=0}^{n}{B}_{1,i}-f{H}_{1}{\sum }_{i=0}^{n}{B}_{1,i},\\ \frac{d{B}_{1,0}}{dt} & = & (q-1){B}_{1,0}({\sigma }_{c}{G}_{1}+{\sigma }_{c}{G}_{2})-{d}_{B}{B}_{1,0}+{\alpha }_{0}(1-p-d-q){B}_{1,1}({\sigma }_{c}{G}_{1}+{\sigma }_{c}{G}_{2}),\\ \frac{d{B}_{1,i}}{dt} & = & p{\alpha }_{i}{B}_{1,(i-1)}({\sigma }_{c}{G}_{1}+{\sigma }_{c}{G}_{2})+(q-1){B}_{1,i}({\sigma }_{c}{G}_{1}+{\sigma }_{c}{G}_{2})-{d}_{B}{B}_{1,i}+{\alpha }_{i}(1-p-d-q){B}_{1,i+1}({\sigma }_{c}{G}_{1}+{\sigma }_{c}{G}_{2}),\\ \frac{d{B}_{1,k}}{dt} & = & p{\alpha }_{k}{B}_{1,(k-1)}({\sigma }_{c}{G}_{1}+{\sigma }_{c}{G}_{2})+(q-1){B}_{1,k}({\sigma }_{c}{G}_{1}+{\sigma }_{c}{G}_{2})-{d}_{B}{B}_{1,k}-\kappa {B}_{1,k}+{\alpha }_{k}(1-p-d-q){B}_{1,k+1}({\sigma }_{c}{G}_{1}+{\sigma }_{c}{G}_{2}),\\ \frac{d{B}_{1,n}}{dt} & = & p{\alpha }_{n}{B}_{1,(n-1)}({\sigma }_{c}{G}_{1}+{\sigma }_{c}{G}_{2})-{d}_{B}{B}_{1,n}-\kappa {B}_{1,n}+(q-1){B}_{1,n}({\sigma }_{c}{G}_{1}+{\sigma }_{c}{G}_{2})+(1-q-p-d){\alpha }_{n}{B}_{1,n}({\sigma }_{c}{G}_{1}+{\sigma }_{c}{G}_{2}),\\ \frac{d{P}_{1}}{dt} & = & {\sum }_{k={n}_{c}}^{n}{\alpha }_{k}\kappa {B}_{1,k},\\ \frac{d{G}_{2}}{dt} & = & -{d}_{G}{G}_{2}-{G}_{2}{\sum }_{i=0}^{n}(\eta {B}_{1,i}+\eta {B}_{2,i})+{H}_{2}{\sum }_{i=0}^{n}(f{B}_{1,i}+f{B}_{2,i}),\\ \frac{d{H}_{2}}{dt} & = & -{d}_{G}{H}_{2}+{G}_{2}{\sum }_{i=0}^{n}(\eta {B}_{1,i}+\eta {B}_{2,i})-{H}_{2}{\sum }_{i=0}^{n}(f{B}_{1,i}+f{B}_{2,i}),\\ \frac{d{B}_{2,0}}{dt} & = & (q-1)\sigma {B}_{2,0}{G}_{2}-{d}_{B}{B}_{2,0}+{\alpha }_{0}(1-p-d-q){B}_{2,1}\sigma {G}_{2},\\ \frac{d{B}_{2,i}}{dt} & = & p{\alpha }_{i}\sigma {B}_{2,(i-1)}{G}_{2}+(q-1)\sigma {B}_{2,i}{G}_{2}-{d}_{B}{B}_{2,i}+{\alpha }_{i}(1-p-d-q){B}_{2,i+1}\sigma {G}_{2},\\ \frac{d{B}_{2,k}}{dt} & = & p{\alpha }_{k}\sigma {B}_{2,(k-1)}{G}_{2}+(q-1)\sigma {B}_{2,k}{G}_{2}-{d}_{B}{B}_{2,k}-\kappa {B}_{2,k}+{\alpha }_{k}(1-p-d-q){B}_{2,k+1}\sigma {G}_{2},\\ \frac{d{B}_{2,n}}{dt} & = & p{\alpha }_{n}\sigma {B}_{2,(n-1)}{G}_{2}-{d}_{B}{B}_{2,n}-\kappa {B}_{2,n}+(q-1){B}_{2,n}\sigma {G}_{2}+(1-q-p-d){\alpha }_{n}{B}_{2,n}\sigma {G}_{2},\\ \frac{d{P}_{2}}{dt} & = & {\sum }_{k={n}_{c}}^{n}{\alpha }_{k}\kappa {B}_{2,k},\end{array}$$

with initial conditions ${G}_{j}(0)={G}_{{j}_{0}}$, *H*_*j*_(0) = 0, ${B}_{j,0}(0)={B}_{{j}_{0}}$, *B*_*j*,*i*_(0) = 0, *P*_*j*_(0) = 0, where *j* ∈ {1, 2} represents the clone, *i* ∈ {1, …, *n*_*c*_ − 1} represents the selection stage that does not result in plasma cell output, and *k* ∈ {*n*_*c*_, …, *n*} represents the selection stage that results in plasma cell output. All simulations are completed in Matlab2017a^30^ using the ode45 ode solver. All code is available on github (https://github.com/laurenchilds/GC).

Box 1: Terminology *Tfh family*: Describes the individual classes of T follicular helper cells: the available subpopulations drive the selection of B-cells of strain-specific or broadly reactive B-cell clones into highly mutated cells inside a germinal center.

*B clone*: Describes the collection of B-cells of all mutational stages with a determined specificity to antigen and Tfh-cells. Each clone is comprised of *n* stages.

*Stage*: Refers to one mutational step of a cell in a B-cell clone in the course of the germinal center reaction. Each subsequent stage represents a productive somatic hypermutation that has been selected.

### Parameters

Parameter values from previously published papers are utilized here as follows. We assume Tfh-cells live on average 100 days and, thus, die at rate, *d*_*G*_ = 0.01 per day^31^, and all B-cells in the germinal center live on average 30 hours or die at rate *d* = 0.8 per day^32^. We assume plasma cells are formed at rate *κ* = 1.2 per day, by all *B*_*k*_ cells passing a threshold selection stage *k* ≥ *n*_*c*_, with *n*_*c*_ = 2∕3 ⋅ *n*. B-cells produce on average *α*_*i*+1_ = 8 ⋅ (1 + *i*∕*n*) offspring during each forward mutation, such that *α*_0_ = *α*_1_ = 8 per day and *α*_*n*_ = 16 per day^33^. We define *σ* as the per Tfh-cell selection rate of B-cell mutational stages, and the combination *α*_*i*_*σG* as the productive somatic hypermutation rate. For the strain-specific selection rate, we use a baseline value of 1.7 ⋅ 10^−4^ ml per cell per day, larger than in^29^. The four different events considered during each stage of somatic hypermutation are forward mutation with probability *p* = 0.18, deleterious mutation with probability *d* = 0.3, neutral mutation with probability *q* = 0.5 and backwards mutation with probability 1 − *p* − *q* − *d* = 0.02^34,35^. The decrease in Tfh availability during germinal center affinity maturation occurs at rate *η*, which accounts for competition between the B-cells for Tfh-cell signal, and has a value of 10^−5^ per cell per day. The regain in Tfh availability during germinal center affinity maturation occurs at rate *f*, and has a value of 10^−5^ per cell per day. We expect that the broadly reactive B-cell selection rate per Tfh-cell, *σ*_*c*_, is lower than the strain-specific B-cell selection rate per Tfh-cell, *σ*, as Tfh families provide broadly reactive B-cell clones with less support for B-cell activation, class switching and somatic hypermutation. The overall broadly reactive B-cell selection, *σ*_*c*_(*G*_1_ + *G*_2_), however, can be higher than the overall strain-specific B-cell selection, *σG*_2_. The broadly reactive selection rate, *σ*_*c*_, is varied, relative to the strain-specific selection rate, *σ*, throughout the paper. Parameter values and initial conditions are summarized in Table 1.

**Table 1:** Parameter values and initial conditions. The per Tfh-cell broadly reactive selection rate *σ*_*c*_ and the initial B-cell clone values are adjusted throughout the study.

Parameter	Description	Units	Baseline Value	Ref
*n*	B-cell clone somatic hypermutation stage	—	8 (50)	^29^
*n*_*c*_	somatic hypermutation threshold for plasma production	—	2∕3 ⋅ *n*	
*η*	Tfh-cell loss of availability rate during germinal center affinity maturation	cell^−1^ day^−1^	10^−5^	^29^
*f*	Tfh-cell regain of availability rate during germinal center affinity maturation	cell^−1^day^−1^	10^−5^	
*σ*	per Tfh-cell strain-specific B-cell selection rate	ml cell^−1^day^−1^	1.7 ⋅ 10^−4^	
*σ*_*c*_	per Tfh-cell broadly reactive B-cell selection rate	ml cell^−1^ day^−1^	0.5*σ*	
*p*	forward mutation		0.18	^34,35^
*d*	deleterious mutation		0.3	^34,35^
*q*	neutral mutation		0.5	^34,35^
1 − *p* − *d* − *q*	backward mutation		0.02	^34,35^
*d*_*G*_	Tfh-cell death rate	day^−1^	0.01	^31^
*d*_*B*_	B-cell death rate	day^−1^	0.8	^37^
*κ*	Plasma cell production rate	day^−1^	1.2	
*α*_*i*+1_	*B*_*i*_ -cell proliferation	—	8 ⋅ (1 + *i*∕*n*)	^33^
*G*_1_(0)	Initial number of available Tfh-cells	cells/ml	5000	
*G*_2_(0)	Initial number of available Tfh-cells	cells/ml	5000	
*H*_1_(0)	Initial number of unavailable Tfh-cells	cells/ml	0	
*H*_2_(0)	Initial number of unavailable Tfh-cells	cells/ml	0	
*B*_2,0_(0)	Initial number of *B*_1_-cells	cells	50	^9^
*B*_2,0_(0)	Initial number of *B*_2_-cells	cells	50	^9^

In order for our continuous model formulation, where clones cannot disappear in finite time, to capture the biological phenomenon of out competing other clones, we must set a threshold for what is considered success. In line with previous studies, we define a plasma output of more than 100 cells as a successful germinal center^14,36^. We consider a single B-cell clone to be successful if it produces at least 100 plasma cells from the germinal center. Although cells of both clones may exist in a germinal center, we refer to a germinal center as monoclonal if only cells of one clone have produced plasma cells above our threshold.

## Results

### Factors that influence the composition of germinal centers

Our study assumes that B-cells from two different B-cell clones seed a germinal center: Tfh-cell broadly reactive and Tfh-cell strain-specific. The characteristics of each B-cell clone has a potential influence on overall B-cell selection and expansion, as well as B-cell clonal dominance, and even competitive exclusion of other B-cell clones or B-cell types. We are interested in determining which factors influence outcomes, such as germinal center size, time till termination, plasma cell numbers, and most importantly, the types of plasma cells that dominate an immune response following germinal center completion. To determine that, we investigate how early B-cell clone seeding, germinal center size, and the total number of rounds of productive somatic hypermutations needed for plasma cell production correlate with different outcomes.

Most infections lead to an average of 5–10 rounds of somatic hypermutations before antibody gets produced; such antibody may or may not be able to contain the infection^38,39^. Some chronic infections, however, require as many as 90 rounds of somatic hypermutations before an antibody is produced (see, for example, VRC01 in HIV infections^40^). We aim to understand the mechanistic interactions that lead to germinal centers where one B-cell clone out-competes the other or where the two B-cell clones coexist. We investigate this in the context of plasma (antibody) production requiring low and high number of productive somatic hypermutations, and we focus on the total plasma cell composition (rather than B-cell clone composition).

### Germinal center dynamics following few mutational stages

We model germinal center responses with a low number of selection stages by assuming *n* = 8 in model (1)^29^. Here, we assume that each selection stage corresponds with a beneficial (forward or backward) mutation, which may require multiple rounds of somatic hypermutation. We assume *B*_1,0_(0) = *B*_2,0_(0) = 50 cells, and that a total initial Tfh-cell population is equally distributed between the two Tfh-cell families, i.e. *G*_1_(0) = *G*_2_(0) = 5000 cells per ml. We choose *σ* = 1.7 ⋅ 10^−4^ ml/(cell ⋅ day) and *η* = 10^−5^ /(cell ⋅ day) motivated from previous work^29,33^. As higher affinity B-cells replicate faster, we increase replication from 8 hours per replication (8x) to 6 hours per replication (16x) as *α*_*i*+1_ = 8 ⋅ (1 + *i*∕*n*).

#### Equal seeding of B-cell clones to initiate the germinal center

**Figure 1:**
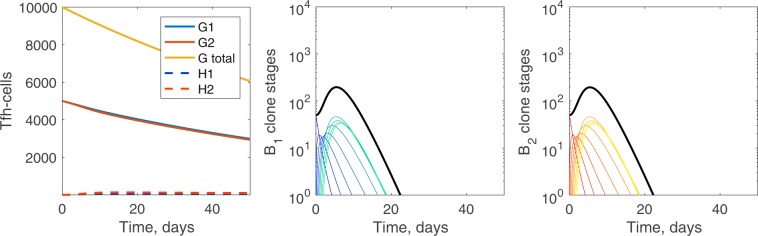
Germinal center dynamics for few required mutational stages. Tfh- and B-cell dynamics over the course of the germinal center reaction for *n* = 8 mutational stages: (left) Tfh-cells, (center) broadly reactive *B*_1_ clone mutational stages, and (right) strain-specific *B*_2_ clone mutational stages. Blue lines are related to B-cells from clone 1 and red lines to B-cells from clone 2. Lighter colors of B-cells refer to later mutational stages. The solid black line in the B-cell panels is the total population of all mutational stages. Parameters and initial conditions are *η* = 10^−5^, *σ* = 1.7 ⋅ 10^−4^, *σ*_*c*_ = 0.5*σ*, *B*_1,0_(0) = *B*_2,0_(0) = 50, *G*_1_(0) = *G*_2_(0) = 5000, *H*_1_(0) = *H*_2_(0) = 0.

Initially, we assume that the seeding B-cells are equally distributed between the two B-cell clones, *B*_1,0_(0) = *B*_2,0_(0) = 50 cells, and that the per Tfh-cell broadly reactive selection rate is half the per Tfh-cell strain-specific selection rate, *σ*_*c*_ = 0.5*σ*. In Fig. 1, the progression of B-cell mutational stages inside each clone shows nearly symmetrical usage of Tfh-cells by the two B-cell clones. The germinal center terminates after just over 22 days when the death and deleterious mutation of B-cells exceeds selection to transition to the next mutational stage.

**Figure 2:**
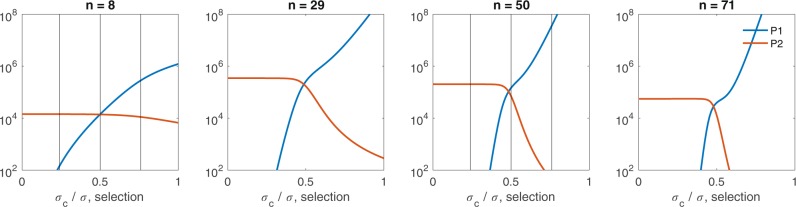
Plasma cell output as *σ*_*c*_ is varied relative to *σ*. Maximum number of mutational stages (left) *n* = 8, (middle left) *n* = 29, (middle right) *n* = 50, (right) *n* = 71, alters the plasma cell population for both broadly reactive *B*_1_ clone (blue) and strain-specific *B*_2_ clone (red). Plasma cell production occurs for *n* > *n*_*c*_ stages, where ${n}_{c}=\frac{2}{3}n$. Vertical black lines in the left panel and middle right panel correspond to the results of *σ*_*c*_*/σ* ratios, i.e. *σ*_*c*_ ∈ {0.24, 0.5, 0.76}*σ*, shown in Fig. 6. An equal fraction of each B-cell clone seeds the germinal center. Other parameters and initial conditions are *η* = 10^−5^, *σ* = 1.7 ⋅ 10^−4^, *B*_1,0_(0) = *B*_2,0_(0) = 50, *G*_1_(0) = *G*_2_(0) = 5000, *H*_1_(0) = *H*_2_(0) = 0.

**Figure 3:**
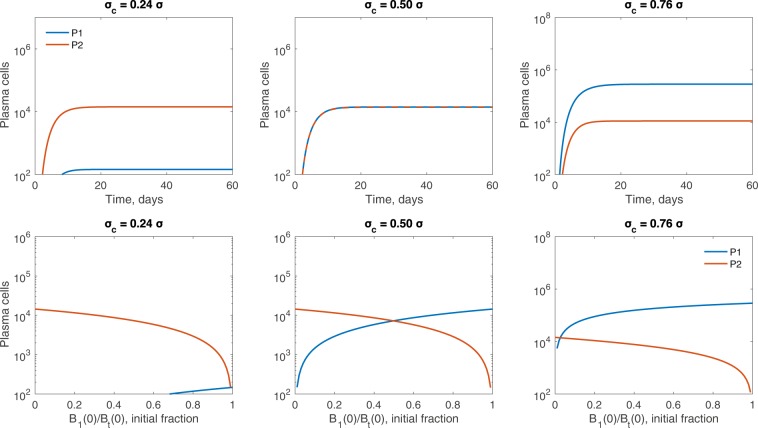
Germinal center outcomes for few required mutational stages. Top row: Plasma cell dynamics over time for *n* = 8. Bottom row: Plasma cell amounts at germinal center termination over the initial *B*_1_(0)∕*B*_*t*_(0) ratio for *n* = 8. The per Tfh-cell broadly reactive selection rates *σ*_*c*_ are varied with (left) *σ*_*c*_ = 0.24*σ*, (middle) *σ*_*c*_ = 0.5*σ*, and (right) *σ*_*c*_ = 0.76*σ*. Other parameters and initial conditions are *η* = 10^−5^, *σ* = 1.7 ⋅ 10^−4^, *G*_1_(0) = *G*_2_(0) = 5000, *H*_1_(0) = *H*_2_(0) = 0.

We are interested in the overall composition of the plasma cell population when the per Tfh-cell broadly reactive selection rate *σ*_*c*_ is varied. For *n* = 8 and equal seeding, model (1) predicts successful production of plasma cells of both types, broadly reactive *P*_1_ and strain-specific *P*_2_ (defined as a population containing ≥ 100 cells) when *σ*_*c*_ > 0.24*σ* (see Fig. 2, left panel). For *σ*_*c*_ < 0.24*σ*, only *P*_2_, plasma cells of the strain-specific *B*_2_ clone, are found. When both plasma types are produced, dominance is determined by the size of *σ*_*c*_. In particular, plasma cells produced by the strain-specific B-cell clone, *P*_2_, dominate when *σ*_*c*_ < 0.5*σ*, due to overall faster selection rate in the *B*_2_ population given by *σG*_2_ (see Fig. 3, top left panel, for an example where *σ*_*c*_ = 0.24*σ*). When *σ*_*c*_ = 0.5*σ* with equal seeding, where the B-cells in both clones are nearly identical as shown in Fig. 1, we see comparable amounts of plasma cells formed from both B-cell clones (see Fig. 3, top center panel). Finally, when *σ*_*c*_ > 0.5*σ*, *P*_1_ family dominates due to overall larger selection rate in the broadly reactive *B*_1_ population as given by *σ*_*c*_*G*_1_ + *σ*_*c*_*G*_2_, as compared to only *σG*_2_ selection in the *B*_2_ population (see Fig. 3, top right panel, for an example where *σ*_*c*_ = 0.76*σ*).

#### Biased seeding of B-cell clones to initiate the germinal center

Experimental studies have suggested that the homogeneity of the germinal centers requires rapid selection and expansion early on of B-cells from the same clone^9^. We test this hypothesis by varying the initial cell ratios in the two B-cell clones, *B*_*i*_(0)∕*B*_*t*_(0) ∈ (0, 1), while keeping an equal amount of available Tfh-cells for each family, *G*_1_(0) = *G*_2_(0) = 5000. In the bottom row of Fig. 3, we plot the amount of cells in the broadly reactive *P*_1_ and strain-specific *P*_2_ populations following the termination of germinal centers as we vary the initial proportion *B*_*i*_(0)∕*B*_*t*_(0) considering three different per Tfh-cell broadly reactive selection rates, *σ*_*c*_. As in the unbiased seeding case, we get successful production of plasma cells regardless of *B*_*i*_(0)∕*B*_*t*_(0) ∈ (0, 1) and *σ*_*c*_ ∈ {0.24, 0.5, 0.76}. In addition, both plasma cell types are produced for *σ*_*c*_ ∈ {0.24, 0.5, 0.76}. Plasma type dominance for the three *σ*_*c*_ values considered, however, can be shifted based on the initial seeding. For example, for *σ*_*c*_ = 0.24*σ* the broadly reactive *P*_1_ population dominates when at least 99% of the seeding B-cells come from clone 1 (see Fig. 3, bottom left). This contrasts with earlier appearance and overall dominance by the strain-specific *P*_2_ population for equal seeding (see Fig. 3, top left). Conversely, for *σ*_*c*_ = 0.76*σ*, broadly reactive *P*_1_ population is dominant when as few as 3% of the seeding B-cells come from the broadly reactive clone (see Fig. 3, bottom right), in line with overall dominance for equal seeding (see Fig. 3, top right). Lastly, for *σ*_*c*_ = 0.5*σ*, the broadly reactive *P*_1_ population is dominant when at least 50% of the seeding B-cells come from the broadly reactive clone (see Fig. 3, bottom center), as seen by nearly identical appearance and dynamics for equal seeding (see Fig. 3, top center).

The relative *σ*_*c*_∕*σ* ratio has an effect not only on the composition of the overall plasma population, but also its magnitude. For *n* = 8, the overall plasma magnitude at the end of the germinal center increases as *σ*_*c*_∕*σ* increases (see Fig. 2, left panel). This occurs due to rapid selection of B-cells from broadly reactive *B*_1_ clone in the presence of sufficient help provided by the two Tfh-cell families. The relative increase of *σ*_*c*_ has an opposite, but, importantly, not as strong, effect on the strain-specific *B*_2_ clone. Competition reduces the production of *P*_2_ plasma cells.

To check whether the magnitude and/or composition of the germinal centers are affected when the Tfh-cell help is insufficient, we plot both plasma populations at the germinal center termination, for higher number of mutations *n* = 29, *n* = 50 and *n* = 71. These are representative of infections where increased rounds of selection are needed for production of potent plasma cells (see, for example, VRC01 in HIV infections^40^). Interestingly, the overall magnitude of the plasma cell population and the qualitative outcome (monoclonal or biclonal germinal centers) varies non-monotonically with *n* and *σ*_*c*_∕*σ* (see Fig. 2). In cases where fewer mutational stages are required to produce plasma cells, increases in *σ*_*c*_ result in early but lower levels of broadly reactive plasma cells. When more mutational stages are necessary before plasma production, production of broadly reactive plasma cells is delayed and requires larger *σ*_*c*_. The broadly reactive plasma population increases as *σ*_*c*_ increases (see Fig. 2, *n* = 29, *n* = 50 and *n* = 70). This is the result of intraclonal competition for Tfh-cells, which forces the selection, *σ*_*c*_(*G*_1_ + *G*_2_), below the B-cell death rate. When more mutational stages are required, exponentially more B-cells are produced, increasing competition. The strain specific plasma population decays to undetectable levels, with the time to non-detection occurring for smaller *σ*_*c*_ as *n* increases. This is the result of interaclonal competition for Tfh-cells.

To determine the mechanisms responsible for the germinal center limited growth and/or termination before reaching the production of plasma cells at *n*_*c*_ mutational stages for higher *n*, we will focus on the *n* = 50 case.

### Germinal center dynamics with many mutational stages

#### Equal seeding of B-cell clones to initiate the germinal center

**Figure 4:**
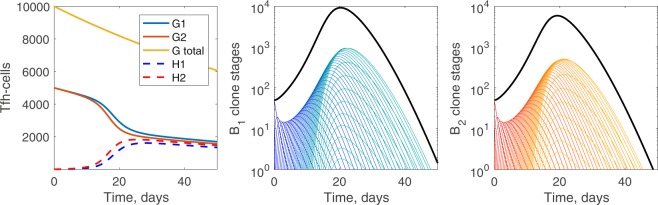
Germinal center dynamics for many required mutational stages. For *n* = 50, (left) Tfh-cells, (center) broadly reactive *B*_1_ clone mutational stages, and (right) strain-specific *B*_2_ clone mutational stages. Blue lines are related to B-cell clone 1 and red lines to B-cell clone 2. Lighter colors of B-cells refer to later mutational stages. The solid black line in the B-cell panels is the total population of all mutational stages. Parameters and initial conditions are *η* = 10^−5^, *σ* = 1.7 ⋅ 10^−4^, *σ*_*c*_ = 0.5*σ*, *B*_1,0_(0) = *B*_2,0_(0) = 50, *G*_1_(0) = *G*_2_(0) = 5000, *H*_1_(0) = *H*_2_(0) = 0.

In Fig. 4, we present the temporal progression of B-cells and Tfh-cells for *n* = 50. There is asymmetrical usage of Tfh-cells, with the Tfh-cell population used by the broadly reactive B-cell clone falling slightly lower. As in the case of fewer selectional stages, the germinal center terminates because the death of B-cells and the deleterious mutations exceed the selection. Although the Tfh-cell population has been significantly diminished, the population is not yet exhausted.

**Figure 5:**
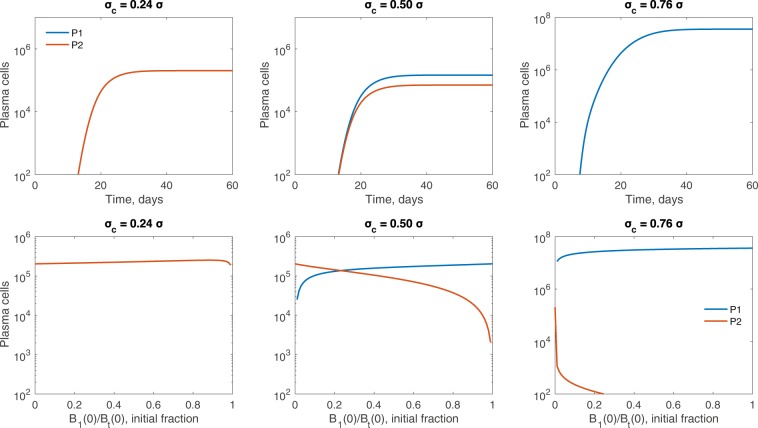
Germinal center outcomes for many required mutational stages. Top row: Plasma cell dynamics over time for *n* = 50. Bottom row: Plasma cell amounts at germinal center termination over the initial *B*_1_(0)∕*B*_*t*_(0) ratio for *n* = 50. The per Tfh-cell broadly reactive selection rates *σ*_*c*_ are varied with (left) *σ*_*c*_ = 0.24*σ*, (middle) *σ*_*c*_ = 0.5*σ*, and (right) *σ*_*c*_ = 0.76*σ*. Other parameters and initial conditions are *η* = 10^−5^, *σ* = 1.7 ⋅ 10^−4^, *G*_1_(0) = *G*_2_(0) = 5000, *H*_1_(0) = *H*_2_(0) = 0.

As in the *n* = 8 case, we are interested in the overall composition of the plasma cell population when the per Tfh-cell broadly specific selection rate *σ*_*c*_ is varied. For *n* = 50 and equal seeding, model (1) predicts successful production of plasma cells of type *P*_2_ alone when *σ*_*c*_ < 0.38*σ*, *P*_1_ and *P*_2_ when 0.38*σ* < *σ*_*c*_ < 0.62*σ*, and of type *P*_1_ alone when *σ*_*c*_ > 0.62*σ* (see Fig. 2, n=50 case). A zoomed in example for equal seeding and *σ*_*c*_ ∈ {0.24, 0.5, 0.76} is given in the top panels of Fig. 5. We see that for *σ*_*c*_ = 0.24*σ*, only *P*_2_ is produced, or the germinal center is dominated by the strain-specific *B*_2_ family. When *σ*_*c*_ = 0.5*σ*, both types of plasma are produced, albeit the broadly reactive *P*_1_ dominates (see Fig. 5, top center panel). This varies from in the *n* = 8 case, and is attributed to usage of Tfh-cells from the second family (*G*_2_) by both B-cell clones during maturation. Since this results in fewer *G*_2_ Tfh-cells and that is the only resource for strain-specific B-cells (clone 2), it slows its selection. Finally, when *σ*_*c*_ = 0.76*σ*, only *P*_1_ is produced, which is attributed to the higher selection rate for the broadly reactive B-cell clone (see Fig. 5, top right panel).

**Figure 6:**
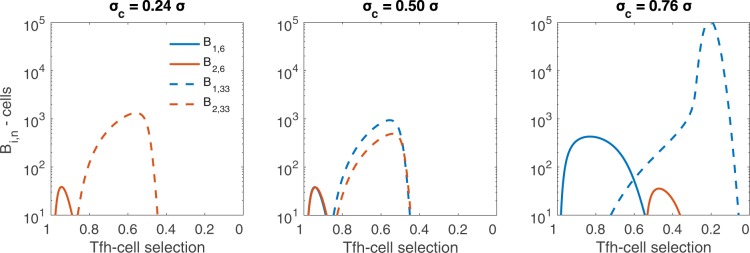
Size of B-cell population in the germinal center as the total selection rates change, for various *σ*_*c*_ values. The size of the ${B}_{i,{n}_{c}-1}$ population for *n* = 8 (solid lines) and *n* = 50 (dashed lines), where *n*_*c*_ − 1 is the last stage before plasma cell formation occurs, is shown for broadly reactive *B*_1_ clone (blue) and strain-specific *B*_2_ clone (red). Note that for *σ*_*c*_ = 0.5*σ*, the *B*_*i*,6_ populations overlap. The x-axis represents *σ*_*c*_(*G*_1_ + *G*_2_) for the *B*_1_ clone and *σG*_2_ for the *B*_2_ clone and scales left to right from higher values to lower values, since as the germinal center proceeds, the Tfh-cell numbers decrease. As *σ*_*c*_ increases, the initial available help for broadly reactive *B*_1_ clone increases. An equal fraction of each clone seeds the germinal center. The per Tfh-cell broadly reactive selection rates *σ*_*c*_ are varied with (left) *σ*_*c*_ = 0.24*σ*, (middle) *σ*_*c*_ = 0.5*σ*, and (right) *σ*_*c*_ = 0.76*σ*. Other parameters and initial conditions are *η* = 10^−5^, *σ* = 1.7 ⋅ 10^−4^, *B*_1,0_(0) = *B*_2,0_(0) = 50, *G*_1_(0) = *G*_2_(0) = 5000, *H*_1_(0) = *H*_2_(0) = 0.

For *n* = 50, we predict an increase in the overall size of the plasma cell population due to recycling of B-cells in plasma producing stages. At early mutational stages, intraclonal competition for Tfh-cell selection is low, due to the presence of fewer B-cells. As the number of B-cells of both clones increases, so does intraclonal competition (amongst each B-cell clone). Likewise, interclonal competition, particularly the use of *G*_2_-type Tfh-cells by the broadly reactive *B*_1_ clone, alters the growth of the strain-specific *B*_2_ clone. When there is equal seeding by both clones, greater selection of the *B*_1_ clone leads to reduction of the *B*_2_ clone. This is apparent in Fig. 6 as the solid red lines, strain-specific *B*_2_ clone, decrease across the panels for *n* = 8, while the solid blue lines, broadly reactive *B*_1_ clone, increase. These patterns are more evident for *n* = 50 (see Fig. 6, dashed lines). In general, requiring more rounds of mutation reduces the Tfh-cell help per B-cell, i.e. the *B*_*i*,*n*_ population appears in the presence of lower levels of Tfh-cell selection, as seen in Fig. 6 where the dashed curves (*n* = 50) appear to the right of the solid curves (*n* = 8). Interestingly, varying the initial seeding of the germinal center to include more cells from broadly reactive *B*_1_ clone does not always lead to reductions in the strain-specific *B*_2_-type plasma population, as discussed in the next section for *σ*_*c*_ = 0.4*σ* in infections requiring large selection stages *n*.

#### Biased seeding of B-cell clones to initiate the germinal center

**Figure 7:**
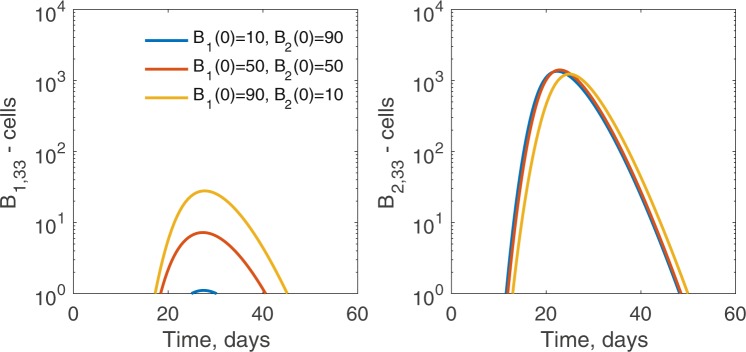
Size of B-cell clones in the germinal center through time, for various inoculum composition. For *σ*_*c*_ = 0.4*σ* and *n* = 50, intraclonal competition in the broadly reactive *B*_1_ clone aids the strain-specific *B*_2_ clone. We plot the size of the ${B}_{i,{n}_{c}-1}$ population, where *n*_*c*_ − 1 is the last stage before plasma cell formation occurs. The *B*_1,33_ population (left panel) increases in magnitude and persists longer as the initial number rises from 10 (blue) to 50 (red) to 90 (yellow) cells. The *B*_2,33_ population (right panel) has non-monotonic changes in magnitude but persists longer as the initial level of *B*_2_ falls from 90 (blue) to 50 (red) to 10 (yellow) cells. Other parameters and initial conditions are *η* = 10^−5^, *σ* = 1.7 ⋅ 10^−4^, *G*_1_(0) = *G*_2_(0) = 5000, *H*_1_(0) = *H*_2_(0) = 0.

In this section, we investigate the effects of biased seeding on plasma cell production for *n* = 50 and *σ*_*c*_ ∈ {0.24, 0.5, 0.76} (see the bottom panel of Fig. 5). When *σ*_*c*_ = 0.24*σ*, we obtain monoclonal germinal centers for all the initial seeding *B*_1_(0)∕*B*_*t*_(0) ratios (see bottom of Fig. 5, left panel). Moreover, the overall magnitude of the plasma cell population at the germinal center termination increases slightly as *B*_1_(0)∕*B*_*t*_(0) increases until a very high *B*_1_ fraction seeds the germinal center. The increase in the plasma cell output is due to interclonal competition where cells from the broadly reactive *B*_1_ clone compete with cells from strain-specific *B*_2_ clone, delaying its growth. This can be more clearly seen for *σ*_*c*_ = 0.4*σ* (see Fig. 7), where the strain-specific *B*_2_ clone undergoes non-monotonic changes in magnitude but each stage persists for longer, allowing larger numbers of cells at later mutational stages, as well as more plasma cells, to be formed. This is a case where weak interclonal competition actually aids the strain-specific *B*_2_ clone. In contrast, when competition is stronger, for example when *σ*_*c*_ = 0.76*σ*, the broadly reactive *B*_1_ clone rapidly out-competes the strain-specific *B*_2_ clone, allowing for little to no growth (see Fig. 5, bottom right panel). This is especially apparent when the germinal center is mainly seeded by the broadly reactive *B*_1_ clone.

When *σ*_*c*_ = 0.5*σ*, we obtain biclonal germinal centers for all initial seeding *B*_1_(0)∕*B*_*t*_(0) (see bottom of Fig. 5, middle panel). As with the equal seeding, however, broadly reactive plasma *P*_1_ dominates the outcome for most initial conditions (see Fig. 5, middle panels).

Lastly, when *σ*_*c*_ = 0.76*σ*, we obtain biclonal germinal centers if the initial seeding *B*_1_(0)∕*B*_*t*_(0) < 0.24, and monoclonal germinal centers producing only broadly reactive *P*_1_ plasma cells when *B*_1_(0)∕*B*_*t*_(0) > 0.24 (see bottom of Fig. 5, right panel). The biclonal germinal centers are composed of a large number of broadly reactive plasma cells *P*_1_, because for *σ*_*c*_ > 0.5*σ* the broadly reactive *B*_1_ clone is selected to a greater extent than the strain-specific *B*_2_ clone. Even when initiating the germinal center from fewer cells, the broadly reactive *B*_1_ clone rapidly out competes the strain-specific *B*_2_ clone for access to Tfh-cell signals.

## Discussion

The mechanistic interactions responsible for the successful production of broadly neutralizing antibodies in response to antigenic challenge take place inside germinal centers where the seeding B-cells undergo proliferation and somatic hypermutation in the presence of antigen and T follicular helper cells. It is generally believed that affinity maturation is driven by competition between cells in the seeding B-cell clones and their somatic mutants, leading to removal of lower affinity B-cells, elimination of entire clones, and, ultimately, production of monoclonal germinal centers. While this may be a common outcome, studies that used multi-photon imaging have shown that multiclonal germinal centers do exist^9,15^. Moreover, elite controllers in chronic infections such as HIV, favor the production of broadly neutralizing antibody obtain from initial selection of low affinity, yet broadly reactive, B-cell clones^17–19^. In this study, we determine the mechanistic interactions leading to monoclonal and biclonal germinal center formation as observed experimentally using multiphoton microscopy^14^. In particular, we investigate the role of early events and Tfh-cell help in germinal center formation and plasma cell production. To account for breadth, we assume that cells from two distinct B-cell clones seed the germinal center. One clone is strain-specific for one Tfh-cell population and the other clone is broadly reactive for both Tfh-cell populations. We investigate the events that lead to monoclonality or biclonality at the germinal center termination, defined as plasma production (at least 100 cells per clone) by one or both B-cell clones, respectively. We find that the results are dependent on the number of mutational stages required prior to plasma cell production and chose two cases, *n* = 8 and *n* = 50, to determine when and why mutational stages matter.

In infections requiring fewer mutational stages for plasma cell production, where an equal number of B-cells from each B-cell clone seed the germinal center, the model results in monoclonal germinal centers that produce strain-specific plasma cells, whenever the per Tfh-cell broadly reactive selection rate is less than one-fourth of the Tfh-cell strain-specific selection rate (Fig. 2 left). Moreover, for higher per Tfh-cell broadly reactive selection rates, biclonal germinal centers are formed, with the dominant plasma cell population depending on the relative size of per Tfh-cell broadly reactive selection rates and initial B-cell seeding. When we assume the per Tfh-cell broadly reactive selection rates are lower than the 24% threshold needed for polyclonality in infections requiring fewer mutational stages, monoclonal germinal centers dominate the outcomes. This is in contrast with experimental results that show polyclonality (at least early in the germinal center reaction) in a large number of germinal centers (9 out of the 12 considered)^15^. In infections requiring fewer mutational stages, we find that, in order to maintain polyclonality for low per Tfh-cell broadly reactive selection rates, the germinal center seeding needs to be biased towards B-cells of the broadly reactive B-cell clone. Tas *et al*. hypothesized that early expansion of B-cells from the same clone leads to homogeneity^9^. Our model agrees with this assumption only if two clones that are strain-specific compete. In that case, the one with the highest initial condition wins and you get a monoclonal germinal center (not shown). However, if strain-specific and broadly reactive clones compete then seeding by a large number of B-cells from the broadly reactive B-cell clone can lead to a biclonal germinal center (Fig. 3, bottom left panel).

In infections requiring more mutational stages prior to plasma cell production, where an equal number of B-cells from each B-cell clone seed the germinal center, we find monoclonal germinal centers consisting only of the strain-specific clone are dominant for an even greater range of per Tfh-cell broadly reactive selection rate (Fig. 2 middle right). As long as the per Tfh-cell broadly reactive selection rate is below 37% of the per Tfh-cell strain-specific selection rate, only the strain-specific clone is successful. Interestingly, a monoclonal germinal center consisting only of the broadly reactive clone can appear when the per Tfh-cell broadly reactive selection rate is greater than 62% per Tfh-cell strain-specific selection rate. Biasing the initial seeding affects the type of outcomes, monoclonal strain specific germinal centers can occur when there is a small concentration of the broad reactive clone and a small selection rate. Monoclonality of the broadly reactive clones occurs when the broadly reactive selection rate is larger. Finally, when the selection rates are similar there is nearly always a polyclonal germinal center in infections with a greater number of mutational stages.

Tfh-cells are considered a limiting resource in germinal center formation^3,14,17– 19,41^. We find that, in the case of low mutational stage infections, the required mutational stages allow for sufficient numbers of cognate Tfh-cells from both Tfh-cell families to be available for selection. Therefore, their influence on the size and composition of a germinal center is negligible. The restrictive role of Tfh-cells in shaping germinal center formation, however, becomes apparent when we increase the mutational stages to account for high mutational stage infections, such as HIV . For *n* = 50, we predict an increase in the overall size of the plasma cell population due to recycling of B-cells in plasma producing stages. However, a high initial number of broadly reactive cells are needed in the inoculum, for the broardly reactive plasma population to emerge. Later in selection the overall B-cell numbers increase as long as Tfh-cell help persists. Vaccination can lead to increased frequency and breadth of Tfh-cells repertoire, hence increased plasma output, as seen in HIV and SIV mosaic vaccination trials^42–45^.

Antigenic variation between pathogen strains such as antigenic drift in influenza^46^ and immune escape in HIV^47^ induces great challenges in designing cross-reactive antibody responses to polyvalent vaccines. Our study did not model antigen and treated the mutating epitopes and/or distinct pathogen strains’ effect on germinal center dynamics to be incorporated in the per Tfh-cell strain-specific and broadly reactive selection rates *σ* and *σ*_*c*_. Under those assumptions, we predict that increased availability of broadly-reactive Tfh-cells (either naturally or induced through vaccination) is needed for inducing immune response breadth. These results may be dependent on the dynamics of the antigen itself. Previous theoretical studies have predicted that direct enhancement and biasing of affinity maturation toward shared or cross-reactive epitopes may form the bases for a successful anti-malaria vaccine^28^. This study did not consider the limiting role of Tfh-cells in germinal center outcomes. Our group has previously shown that when both Tfh-cells and mutating antigen are considered in modeling germinal centers seeded by a single B-cell clone, then the frequency of mutation plays an important role in the observed outcomes. In particular, slow mutating antigen favors breadth while fast mutating antigen favors germinal center termination with limited plasma production^29^. Further work is needed to determine if the same stays true when the germinal center is seeded by multiple B-cell clones.

**Figure 8:**
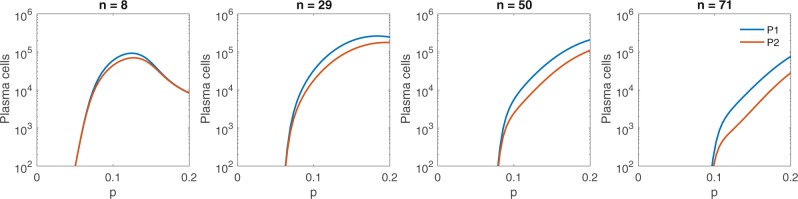
Plasma cell output as the forward mutations vary. Maximum number of mutational stages (left) *n* = 8, (middle left) *n* = 29, (middle right) *n* = 50, (right) *n* = 71 and the fraction of forward mutations, *p*, alter plasma cell populations from both broadly reactive *B*_1_ clone (blue) and strain-specific *B*_2_ clone (red). Plasma cell production occurs for *n* > *n*_*c*_, where ${n}_{c}=\frac{2}{3}n$. An equal fraction of each B-cell clone seeds the germinal center. Other parameters and initial conditions are *η* = 10^−5^, *σ* = 1.7 ⋅ 10^−4^, *B*_1,0_(0) = *B*_2,0_(0) = 50, *G*_1_(0) = *G*_2_(0) = 5000, *H*_1_(0) = *H*_2_(0) = 0.

In this study, we assumed that forward and backward mutations account for 18% and 2%, respectively. To determine if this assumption biases our results, we performed a comparison to determine the effect that advantageous mutations have on the production of plasma populations, *P*_1_ and *P*_2_, for various selection stages, n (see Fig. 8). We find that as *n* increases, the effect of forward mutation rate, *p*, broadens. When *p* is varied in a germinal center with few mutational stages (n=8, Fig. 8), the largest deviation between *P*_1_ and *P*_2_ of 2.3 ⋅ 10^4^ plasma cells occurs when *p* = 0.12. When *p* is varied for germinal centers with increased mutational stages, (n=71, Fig. 8), the largest difference in *P*_1_ and *P*_2_ of 4.8 ⋅ 10^4^ plasma cells occurs at the largest *p* considered, *p* = 0.2.

Our study investigates only two types of B-cell clones and two families of cognate Tfh-cells. This is a limitation that could be extended to increase realism in our results. For example, the low selection range giving rise to monoclonal germinal centers of broad reactivity can be extended by allowing B-cells to receive survival signals from a larger (than the two considered here) repertoire of Tfh-cells or Tfh-cell epitopes, as reported previously^26^. Additionally, we assume a maximum capacity of Tfh-cells initially and, thus, do not model recruitment or expansion of Tfh-cells. Expansion of Tfh-cells was considered in our previous work involving a single B-cell clone^29^; we did not find qualitative differences with the inclusion of expansion (not shown) and thus work from the simplified model. Finally, many parameters are assumed from a previous study that fitted a version of our model to germinal center B-cell data^29^. Since every fitting routine induces uncertainties in the estimated results, our quantitative results are only valid in the context these estimates.

In conclusion, we developed a mathematical model of germinal centers seeded by two B-cell clones and determined the factors that are responsible for the production of monoclonal and biclonal plasma outputs in infections with a differing number of mutational stages prior to plasma cell production.
